# Preparation and Application of Superhydrophobic Copper Mesh by Chemical Etching and *In-situ* Growth

**DOI:** 10.3389/fchem.2021.737550

**Published:** 2021-11-23

**Authors:** Qilei Tong, Zhenzhong Fan, Biao Wang, Qingwang Liu, Yunhe Bo, Liqing Qian

**Affiliations:** ^1^ Department of Petroleum Engineering, Northeast Petroleum University, Daqing, China; ^2^ Qinhuangdao Campus, Northeast Petroleum University, Qinhuangdao, China; ^3^ CNOOC Energy Technology & Services Limited, Tianjin, China

**Keywords:** chemical etching, In situ growth, copper stearate, superhydrophobic copper mesh, oil-water separation

## Abstract

Oily sewage and floating oil in the ocean post a huge threat to the ecological environment, therefore, developing an efficient separation for oil/water mixtures is an urgent need. Currently, superhydrophobic materials exhibit excellent oil/water separation ability. In this study, a superhydrophobic copper mesh prepared by the chemical etching method and the *in-situ* growth method and the performance evaluation are introduced. The oxide layer on the surface of the copper mesh is first removed by pickling, and then immersed in FeCl_3_ solution for chemical etching to make the surface rough, stearic acid (SA) is used for *in-situ* growth to reduce the surface energy, a superhydrophobic oil-water separation copper mesh is obtained. The water contact angle (WCA) of the copper mesh is more than 160°. The copper mesh is chemically stable and can effectively adsorb floating oil and separate the oil-water mixture. After several oil-water separation experiments, the oil-water separation efficiency can still be above 98%. The effects of the concentration of FeCl_3_ and SA on the contact angle and oil-water separation efficiency are investigated, the results show that when the concentration of FeCl_3_ is 2% and SA is 1.5%, the WCA and oil-water separation efficiency are the largest. The research used a simple and environmentally friendly method to prepare the oil-water separation copper mesh, which has important application significance for water quality restoration.

## Introduction

Since the 20th century, due to the intensification of human activities, accidents have occurred in oil transportation or offshore drilling and production platforms, resulting in frequent offshore oil spill accidents (Li and Boufadel, 2010; Ladle et al., 2020). These accidents will not only affect the tourism industry, but also can cause irreversible damage to the ecological environment, and have a fatal threat to the health of human beings, animals and plants (Schwacke et al., 2014; Novakovskiy et al., 2021). With the improvement of environmental awareness, scientists began to focus on how to deal with oil spill problems. In the past decades, controlled combustion (Barnea, 1999; Bitting, 1999; Ghannam and Chaalal, 2003) has become the first choice for many oil spill accidents due to its low cost and no need for complex devices. However, it has a great impact on marine life and environment. In addition it is worth noting that combustion treatment also wastes lots of energy. In order to replace the controlled combustion, chemical dispersion, oil coagulant, filtration method and adsorption method are proposed.

Chemical dispersion shows that oil spill dispersant can promote the rapid emulsification and dispersion of oil spill in water body, and can make the oil degrading microorganisms in the environment more easily and fully contact with the oil spill, then accelerate the natural degradation process of oil spill in water body, which can reduce the concentration of oil spill in a short time to avoid the serious damage of high concentration of oil to the ecological balance of water body. The main limitation of the dispersant is that it is mainly composed of hydrocarbon organic compounds with high toxicity and low biodegradability. When these dispersants contact with water, they are easy to produce toxicity that is harmful to the water environment (Joo et al., 2013; Lee et al., 2013; Zhao et al., 2016).

Scholars have developed a kind of oil coagulant based on chemical dispersant, which can connect the oil to form solid-like carpet, with a slow volume growth rate, and can be easily removed from water, reducing the oil residue and the pollution. With enough high molecular polymers, the spilled oil can become solid eventually. Although the current oil coagulant has good performance, its production cost is high, meanwhile, the existing equipment is designed for recovering crude oil or product oil at present, there is no special recovery device to deal with the residue after oil condensate treatment, therefore, it is still an urgent problem to deal with the residue (Zhuan et al., 2019; Chen et al., 2020; Huang et al., 2020). Although it can be biodegraded (Bragg et al., 1994), this treatment method has high cost and low efficiency.

Compared with the above methods, filtration method and adsorption method are simpler and more feasible, which is easy to achieve continuous oil-water separation, easy to modify for a wide selection of materials, and can be used to separate oil-water mixture containing complex components. The principle of filtration method and adsorption method is to use the superhydrophobic of material to realize oil-water separation.

However, most superhydrophobic materials are difficult to achieve large-scale production in the preparation process, and the separation effect of traditional superhydrophobic oil-water separation materials is not ideal, the low recovery rate and high price restrict their practical application (Wang et al., 2021; Yang et al., 2021; Zhao et al., 2021), Therefore, the development of an efficient and recyclable superhydrophobic system, combined with the recovery of oil production ship, will become the first choice to deal with the oil spill accident in the future (Öner and McCarthy, 2000; Wang et al., 2020; Zeng et al., 2021).

At present, the preparation method of superhydrophobic materials is to roughen the surface of the substrate, and then coat the surface with low surface energy materials. The common methods include laser etching, chemical vapor deposition, template, electrodeposition, chemical etching, etc. Zhang Qian et al. (Zhang and Zhang, 2019) used laser etching and micro arc oxidation technology to construct microstructure on magnesium alloy sheet, and then used fluoride to modify the surface with low surface energy substances, so as to obtain superhydrophobic magnesium alloy sheet with contact angle greater than 160°. Song et al. (2006) used chemical vapor deposition to functionalize the surface of silicon wafer with amino groups, and then modified the surface with fatty acids to change the wettability of the surface. Finally the contact angle of the superhydrophobic surface reached 153°. Sun et al. (2005) used lotus leaf as template and repeatedly poured it with PDMS to obtain the same structure as lotus leaf surface, with contact angle up to 160°. Wang et al. (2017) have obtained superhydrophobic three-dimensional porous foam copper (SOCF) by controlled electro deposition and chemical modification. This is the first time that a three-dimensional porous material with a larger pore size than the emulsion droplet is used to separate the emulsified oil-water mixture. It is proved that the oil-water separation device based on copper can effectively separate various oil-water emulsion, and has high oil flux and amazing wear resistance. Ren Z et al. (2019) used ferric chloride, phosphoric acid and hydrogen peroxide to etch stainless steel wire in two steps to form lotus like micro nano structure on the surface of metal rubber (MR), and then modified by PFDs to obtain water contact angle of 152° The rolling angle is less than 5° It is undeniable that most of the mentioned methods are difficult to achieve large-scale production, the preparation process is more complex, the price of raw materials is high, and some reagents will cause environmental pollution. These shortcomings make them only stay in the laboratory research stage.

In this study, a green superhydrophobic copper mesh was prepared by a simple etching method and *in-situ* growth method. In which, the copper mesh was used as the substrate, the FeCl_3_ was used as chemical etchant and SA that are widely existing in animal fat with low costs was grown *in-situ* on the etched copper mesh to prepare final product. The research significantly improved the shortcomings of the previous oil-water separation copper mesh (complex preparation process/not resistant to acid and alkali corrosion/poor recoverability), and made it widely used in various marine oil spill accidents in the future.

## Experimental

### Materials

Copper mesh(Copper content>99%), which is knitted by wires and the length and width of pore size of the copper mesh is about 75 μm, was obtained from Jiangxi Dali Mesh Co., Ltd., China. Ferric chloride (FeCl_3_) was bought at Shanghai Chemical Reagent Co., Ltd., China. Stearic Acid (SA), Absolute ethanol and other chemicals were commercially available and purchased from Kelong Reagent Co. Ltd., Chengdu, China. All the reagents in this study were of analytical reagent grade, and used without further purification.

### Preparation of Superhydrophobic Copper Mesh

Cut the copper mesh into 10 cm × 10 cm pieces. After soaking in hydrochloric acid for 2 min, the oxide layer on the surface was removed, and then ultrasonic cleaning in distilled water for 10 min and drying. The dried copper mesh was immersed in FeCl_3_ solution with a concentration of 2% for 3 h, and the surface of the mesh was etched by the strong oxidation characteristics of FeCl_3_ to form a rough surface morphology. Then the etched copper mesh was washed with distilled water and dried. The copper mesh was immersed in anhydrous ethanol solution with concentration of 1.5% SA at room temperature for 24 h, then washed with anhydrous ethanol and deionized water twice or three times, and dried at 60°C for 10 min to complete the experiment.

### Superhydrophobic Copper Meshes Characterization

The surface microstructures of the copper mesh were analyzed by a scanning electron microscope, the element analysis was carried out by using the attachment of the scanning electron microscope (Quanta 450 FEG, FEI, United States). In order to determine the valence state of copper after etching, the copper mesh was tested by X-ray photoelectron spectroscopy (XPS, Kratos axis ultra DLD) The static contact angle and dynamic sliding angle were measured *via* a contact angle measurement (CAM200 of KSV Instruments Ltd., Finland). The contact angle to water (WCA) values reported are averages of five measurements made on different points of the sample surface. Deionized water and cyclohexane were used as the main detecting water and oil.

## Results and Discussion

### Surface Morphology Analysis

**FIGURE 1:**
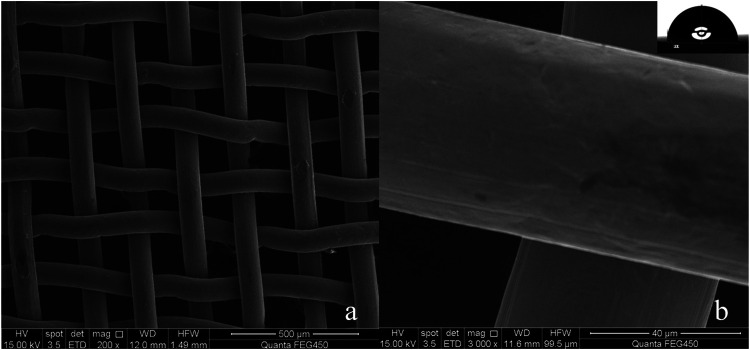
**(A)** Untreated copper mesh (200×), **(B)** Untreated copper mesh (3,000×) and WCA.

Copper mesh is an easily available substrate in industry. Figures 1A,B are the SEM of the surface of the untreated copper mesh. It is smooth and flat (Figure 1B), and many copper wires are interlaced with each other to form many porous structures (Figure 1A). The diameter of these pores is 75 μm, and the whole copper mesh surface presents a micro/nano hierarchical porous structure. The WCA is close to 93° (Figure 1B; Supplementary Video S1. Original copper mesh contact angle video). The contact angle to oil is 0°, the copper mesh is hydrophobic/super-lipophilic.

**FIGURE 2:**
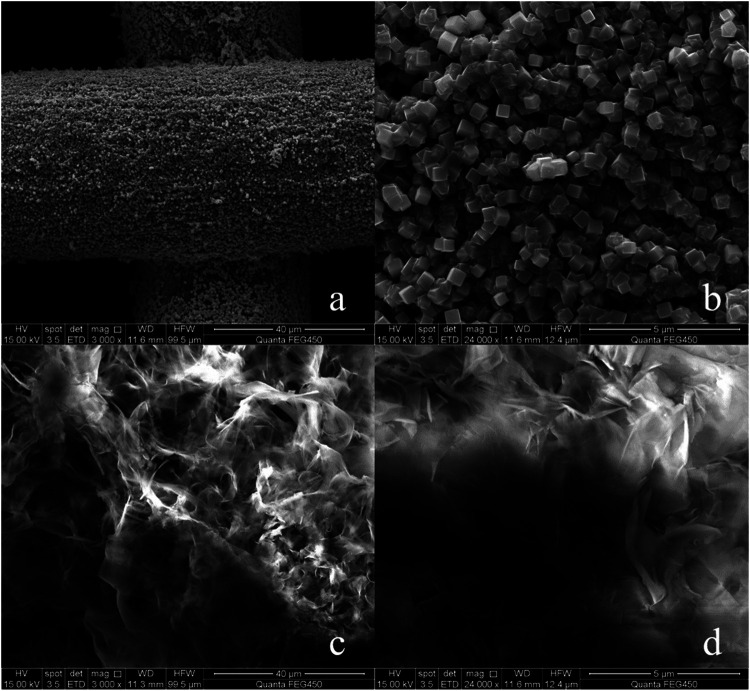
**(A)** FeCl_3_ treated copper mesh (3,000×), **(B)** FeCl_3_ treated copper mesh (240,00×), **(C)** SA modified copper surface (3,000×), **(D)** SA modified copper mesh (240,00×).

In order to make SA grow on the copper mesh *in-situ*, the copper mesh needs to roughened with FeCl_3_. The roughened copper mesh is super-hydrophilic (Supplementary Video S2. Etched copper mesh contact angle video) and the contact angle to oil is still 0°. It can be found that the surface of the copper mesh is covered with a layer of cube shaped particles, and the surface morphology becomes rough (Figures 2A,B). Although the copper mesh becomes rough, the surface of the copper mesh treated with FeCl_3_ only forms CuCl_2_ which can dissolve in water, so the surface shows super-hydrophilic.

After treatment with SA *in-situ* growth method (Figure 2C), the surface of copper mesh is covered with honeycomb structure. From the comparison of Figures 2B,D, it can be seen that the surface reaction between SA and FeCl_3_ treated copper mesh, the copper mesh becomes superhydrophobic.

### Chemical Characterization

**FIGURE 3:**
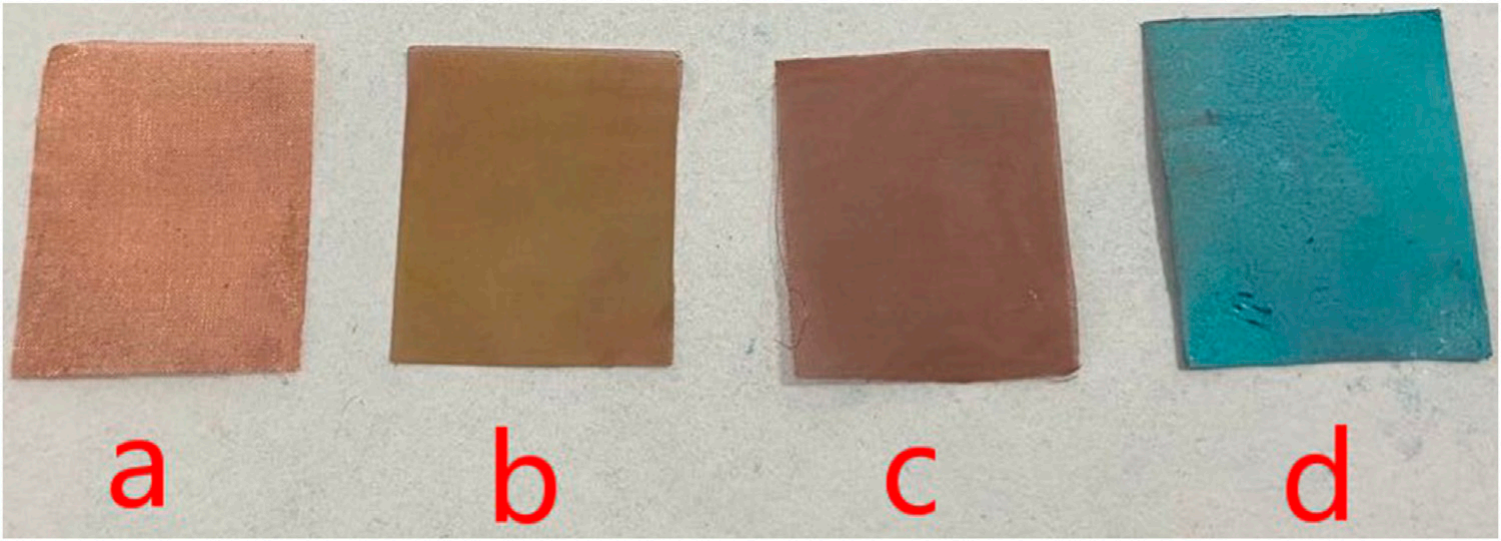
Color change of copper mesh.

Copper mesh has undergone a series of chemical changes during processing. In the first step, the copper mesh is immersed into the hydrochloric acid, mainly to remove organic pollutants and oxides on the surface. The purpose of immersing the copper mesh in the FeCl_3_ solution is to react it with Fe^3+^ which have strong oxidizing properties to form a rough surface on the surface. When the copper mesh is just taken out of the solution, the surface of the copper mesh is yellow green, at this time, the copper mesh surface is CuCl_2_·2H_2_O, after washing and drying, the surface of the copper mesh becomes reddish brown. According to the data, copper chloride appears green in the case of combined water, and anhydrous copper chloride appears brown after drying. It can be seen from Figure 3, it is in line with the information. It means that the rough surface has been formed and the surface brownish substance is copper chloride.

**FIGURE 4:**
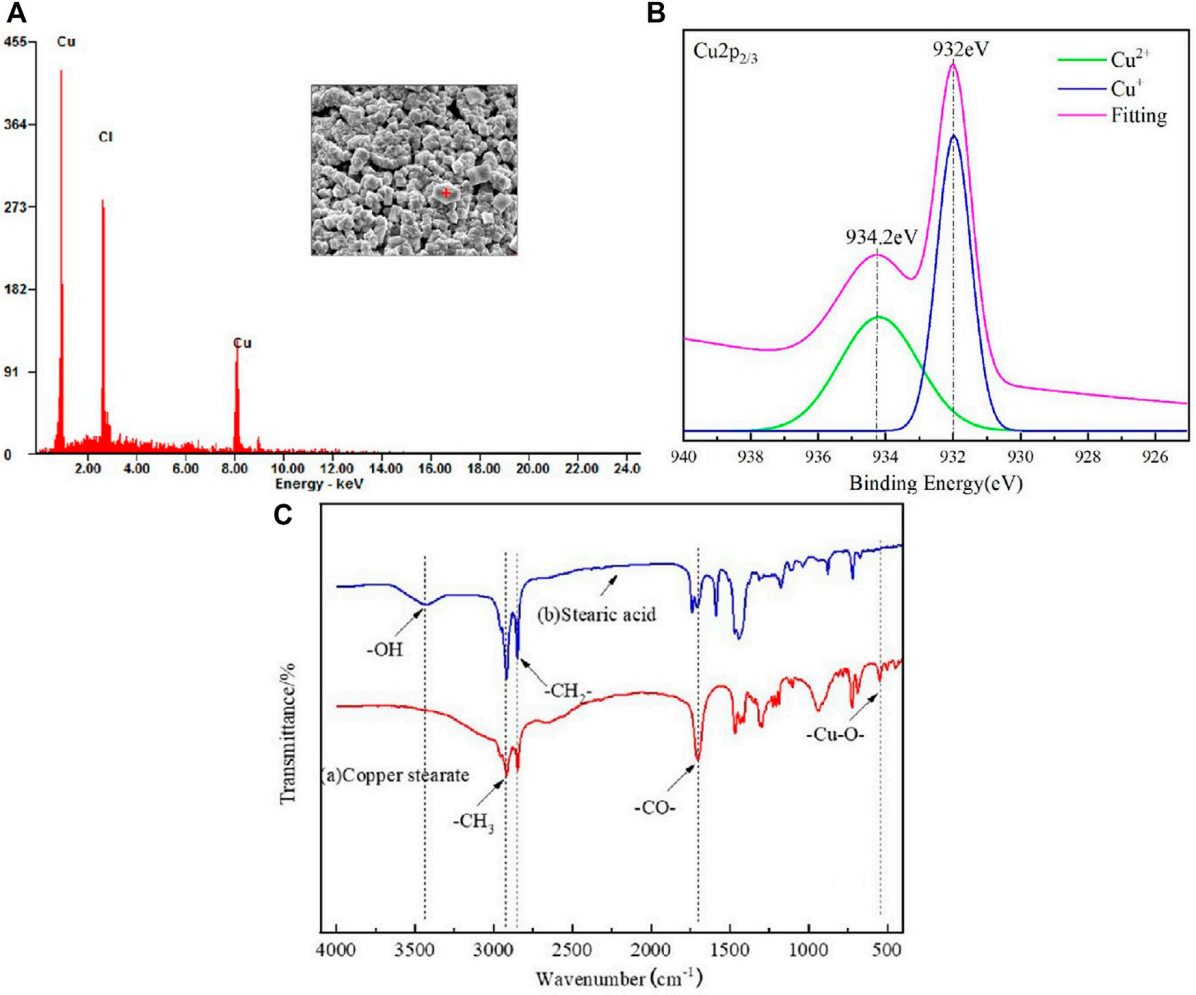
**(A)** EDS spectrum of the FeCl_3_ treated copper mesh surface. **(B)** XPS of the FeCl_3_ treated copper mesh surface.**(C)** FTIR spectra of SA and copper stearate.

EDS was performed to verify surface substance, as shown in Figure 4A. The results display that the massive rough structure of the surface contains Cu and Cl, Fe^3+^ have a strong oxidation ability, and can react with copper elements, so that the copper elements can be converted into Cu^2+^, the chemical reaction equation is as follows(Eq. 1). It is speculated that the reason for two different peaks of copper is that Cu^2+^ is not as stable as Cu^+^, and part of Cu^2+^ becomes Cu^+^ during the drying process(Eq. 2). In order to verify this conjecture, the etched and dried copper mesh is tested by X-ray photoelectron spectroscopy, and the measured electron energy spectrum is fitted by peak(as shown in the Figure 4B), so as to determine the valence state of copper element. According to the analysis of Cu2p_3/2_ spectrum fitting results, 934.2eV is the 3d^10^ peak of divalent copper and 932eV is the 3d^10^ peak of monovalent copper. This result verifies the above conjecture, the dried copper mesh contains both Cu^+^ and Cu^2+^.

$$F{\text{e}}^{3+}+Cu\to Fe^{2+}+Cu^{2+}$$ (1)

$$Cu^{2+\overset{\Delta }{\to }}2Cu^{+}$$ (2)

Superhydrophobic materials need to have two properties, the first one is rough surface, the other one is low surface energy. The interaction of SA and Cu^2+^(Cu^+^) makes the surface of the copper mesh adhere a layer of copper stearate and reduces the surface energy of the copper mesh, so as to obtain superhydrophobic copper mesh. SA reacts with Cu^2+^(Cu^+^) on the surface of copper mesh, and the reaction equation is as follows (Eqs 3, 4).


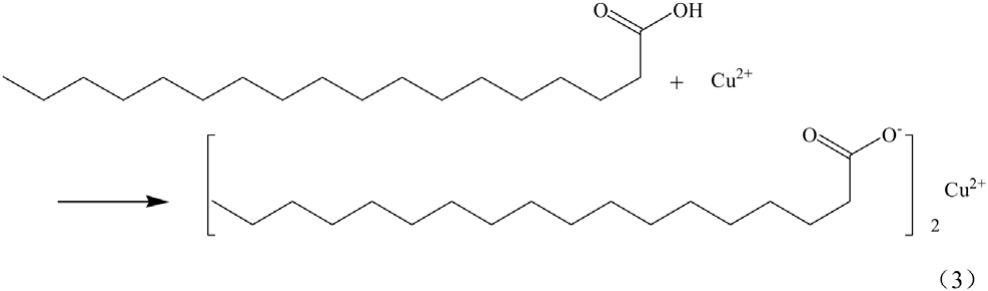
 
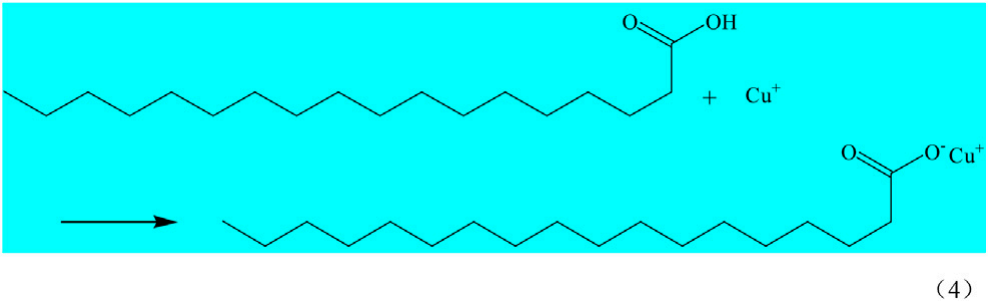


The infrared spectrum of SA and the green attachment on the surface of copper mesh was obtained by KBr pressing method (Figure 4C). In the infrared spectrum, the absorption peak of SA at 3400cm^−1^ is the absorption peak of -OH, while in the infrared spectrum of the green attachment, there is no absorption peak at 3400 cm^−1^, which indicates that -OH has been completely reacted. At 2,923.25 and 2,852.95 cm^−1^, there are vibration absorption peaks of -CH_3_ and -CH_2_-; at 1700 cm^−1^, stearic acid and green powder have absorption peaks of -CO-; at 500 cm^−1^ of green attachment, but stearic acid has no absorption peak, which can be judged as - Cu-O -. Combined with the above analysis and SEM photographs, it can be seen that SA reacts with Cu^2+^ on the surface, the copper stearate was formed.

### Theoretical Analysis of the Effect of Surface Structure on Hydrophobicity

**FIGURE 5:**
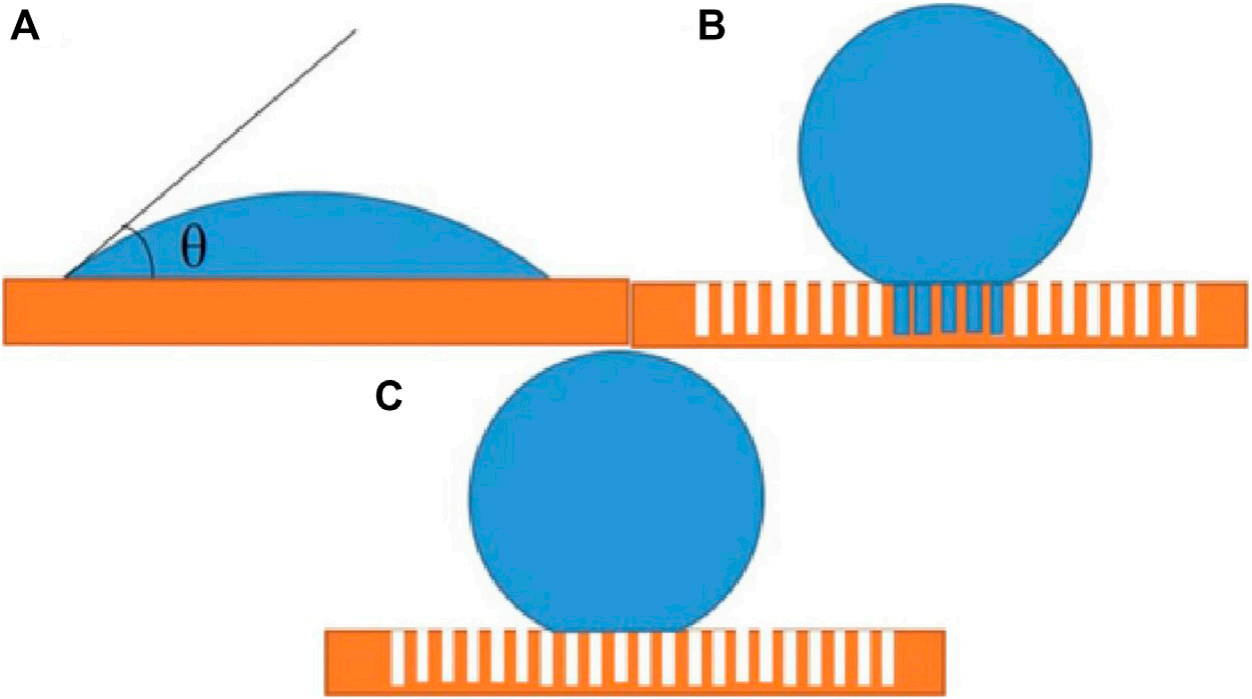
**(A)** Young’s model. **(B)** Wenzel model. **(C)** Cassie and Baxter model.

Wettability of solid surface depends on chemical composition (surface free energy) (Tan et al., 2015) and micro morphology (surface roughness) (Chen et al., 2016). The lower the surface free energy of a solid, the less likely it is to be wetted by a liquid. On the contrary, the higher the surface free energy is, the easier it is to penetrate, and the surface micro-structure can enhance its wettability (He et al., 2019). Therefore, the surface wettability of materials can be changed by surface micro-structure and surface energy. There are several models of solid surface wettability, such as the famous Young’s model (Figure 5A), Wenzel model (Figure 5B) and Cassie Baxter model (Figure 5C) as shown follows:

$$\cos\theta =\frac{\gamma_{sg}-\gamma_{sl}}{\gamma_{\mathrm{lg}}}$$ (5)

cos⁡θ=ωr(γsg−γsl)γlg=r⁡cos⁡θ (6)

$$\cos\theta_{c}=f_{sl}\cos\theta +\left(1-f_{sl}\right)\cos\pi =f_{sl}\cos\theta +f_{sl}-1$$ (7)

“*γ*_*lg,*_ *γ*_*sg,*_ *γ*_*sl*_” is the interfacial tension between gas liquid, solid gas and solid liquid; “*θ*” is intrinsic contact angle (as shown in the Figure 5A), “*θ*_*ω*_” and “*θ*_*c*_” are the actual contact angle between the droplet and the rough surface in the equilibrium state; “r” is defined as the surface roughness coefficient, which is numerically equal to the ratio of the actual area and the projected area of the rough surface; “*f*_*sl*_” is the ratio of the contact area and the total area between the droplet and the solid substrate. Young’s equation (Eq. 5) (Makkonen, 2016) is only applicable to the ideal plane (the surface is completely flat and smooth). However, real solid materials can’t reach the ideal state in terms of chemical composition and geometric structure. In response to the effect of actual rough surface on wettability, Wenzel model (Eq. 6) (Choe et al., 2020; Ciasca et al., 2014) was proposed. When water droplets are on a rough solid substrate, water droplets can enter and fill the grooves in the rough structure. However, there will be a large adhesive force between the droplets and the contact surface, which is not enough to explain the superhydrophobic phenomenon (such as water droplets rolling on the lotus leaf), Therefore, Cassie and Baxter (Eq. 7) (Jiang et al., 2020; Xie and Huang, 2020) assume that the water droplets on the rough surface can’t fill the grooves, thus forming a composite contact, which can better explain some phenomenon.

**FIGURE 6:**
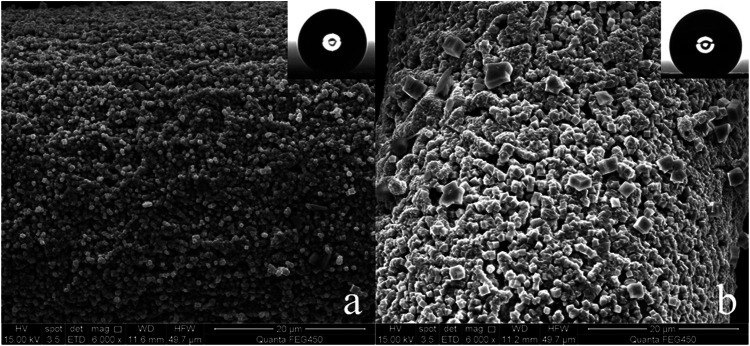
Copper mesh surface with different WCA.

In order to theoretically analyze the influence of superhydrophobic surface microstructure on hydrophobicity, two copper meshes with different WCA are selected (Figure 6), the WCA of a is 164.5° (Figure 6A), the WCA of b is 159° (Figure 6B). Because the substrates are all copper mesh, and they are all *in-situ* grown with a layer of copper stearate, their intrinsic contact angle (θ) is same. As shown in Figure 5, compared with the pictures of two copper meshes etched by FeCl_3_ with the same magnification, the blocky structure on the surface of copper mesh a is obviously smaller and denser than that of copper mesh b, and the rough structure on the surface of copper mesh a is obviously better than that of copper mesh b, when the water droplet contacts on the surface, the contact area of the water droplet on the surface of copper mesh a is obviously smaller than that of b. using the area fraction of f_a_ and f_b_, that is, f_a_ < f_b_, combined with Cassie Baxter equation, it can be seen that the cos θ_a_<cos θ_b_. Therefore, the WCA should be a > b. compared with the theoretical results, the WCA measured above conforms to the theory. Therefore, we can use Cassie Baxter equation to explain the superhydrophobic phenomenon of copper mesh.

### Analysis of Surface Wettability and Surface Energy

**FIGURE 7:**
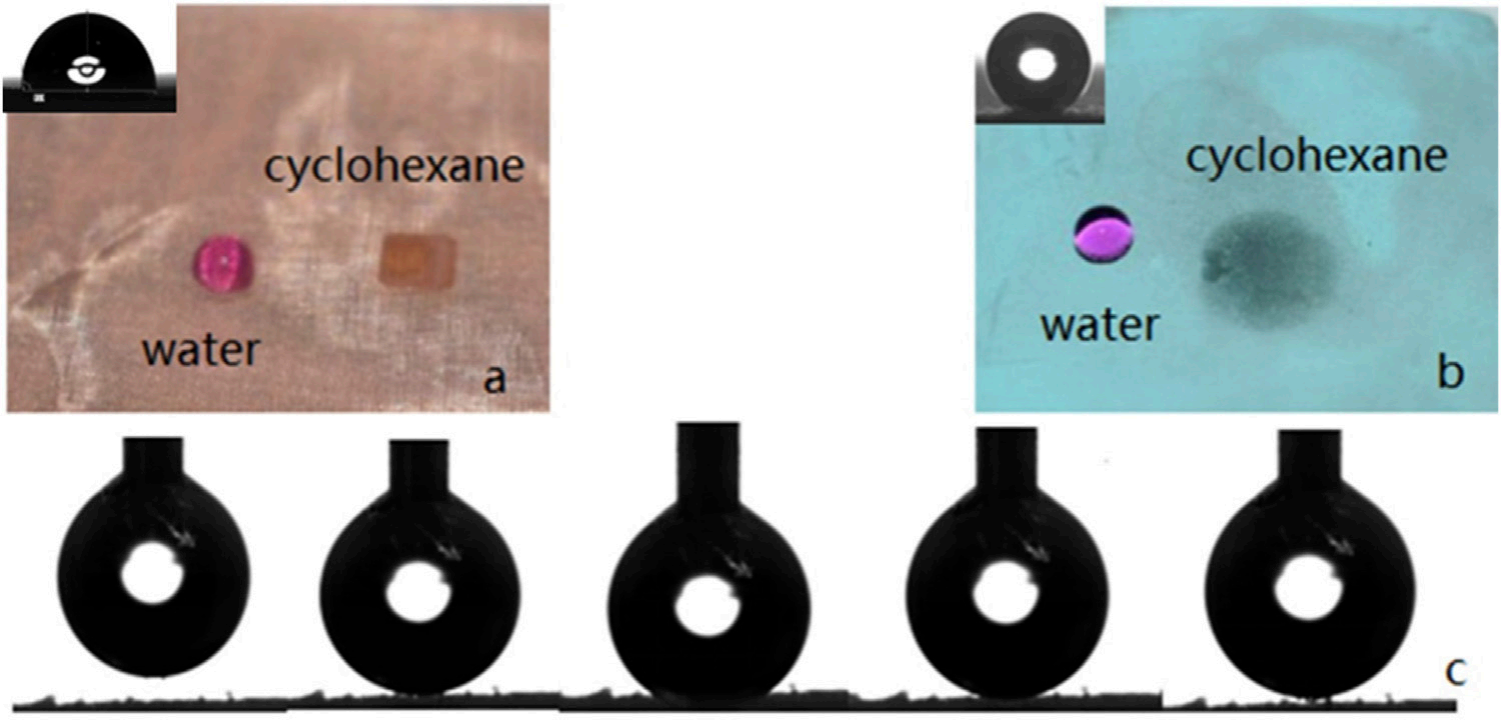
**(A)** The surface of the untreated copper mesh. **(B)** The surface of the treated copper mesh. **(C)** The movement of water droplets on the surface of the copper mesh.

Whether the copper mesh is hydrophobic or not can be determined by measuring the WCA which value is the average of five measurements made at different points on the sample surface. The WCA of untreated copper mesh and treated copper mesh are compared. Figure 7A shows that the surface of the untreated copper mesh is lipophilic because the oil droplets diffuse rapidly on it, while the WCA is 92°, Therefore, the unmodified copper mesh shows certain hydrophobicity and lipophilicity. Figure 7B shows that the oil droplets on the surface of the treated copper mesh can still diffuse rapidly, and the contact angle is close to 0°. However, water appears as a regular sphere on the copper mesh, with a WCA of 164.5° (Supplementary Video S3. Droplets slide on the copper mesh video). The up and down movement of water droplets on the surface of copper mesh is shown in Figure 7C. The water droplets always do not adhere to the surface of copper mesh and the deformation is very small, which indicates that the adhesion of copper mesh to water is low.

As mentioned earlier, the main factors of hydrophobic surface include rough surface and low surface energy materials. Wettability analysis shows that the treated copper mesh has superhydrophobic properties, and the above TEM images have verified that the copper mesh surface has rough structure. To analyze the surface energy of copper mesh before and after treatment, we must first understand the causes of surface energy. The generation of surface energy is due to the asymmetry of the force field of surface atoms (ions and molecules). The nature and asymmetry of surface positions of substances with different structural types are different. The contact angle measurement software can directly calculate the solid surface energy, and its calculation principle is using the Fowkes (Fowkes, 1964) method. (Eq. 8):

$$\gamma_{sg}^{d}=\frac{\gamma_{\mathrm{lg}}{\left(\cos\theta +1\right)}^{2}}{4}$$ (8)

“γ^d^_sg_” is the dispersion force at the solid gas interface, “γ_lg_” is the free energy of liquid gas interface. In 1964, Fowkes proposed to decompose the surface tension into two forces: London dispersion force and polar force consists of couple force, hydrogen bond and induced force. This method finally considers that only the dispersion force acts on the solid-liquid interface, Combined with Young- Dupre equation, the equation is obtained. According to the calculation of contact angle measurement software, the surface energy of copper mesh before treatment is 16.9518 × 10^−3^ J/m^3^, After treatment, the surface energy of copper mesh is 0.0241 × 10^−3^J/m^3^. The calculated results show that the surface energy of copper mesh is greatly reduced after treatment.

### Chemical Stability

**FIGURE 8:**
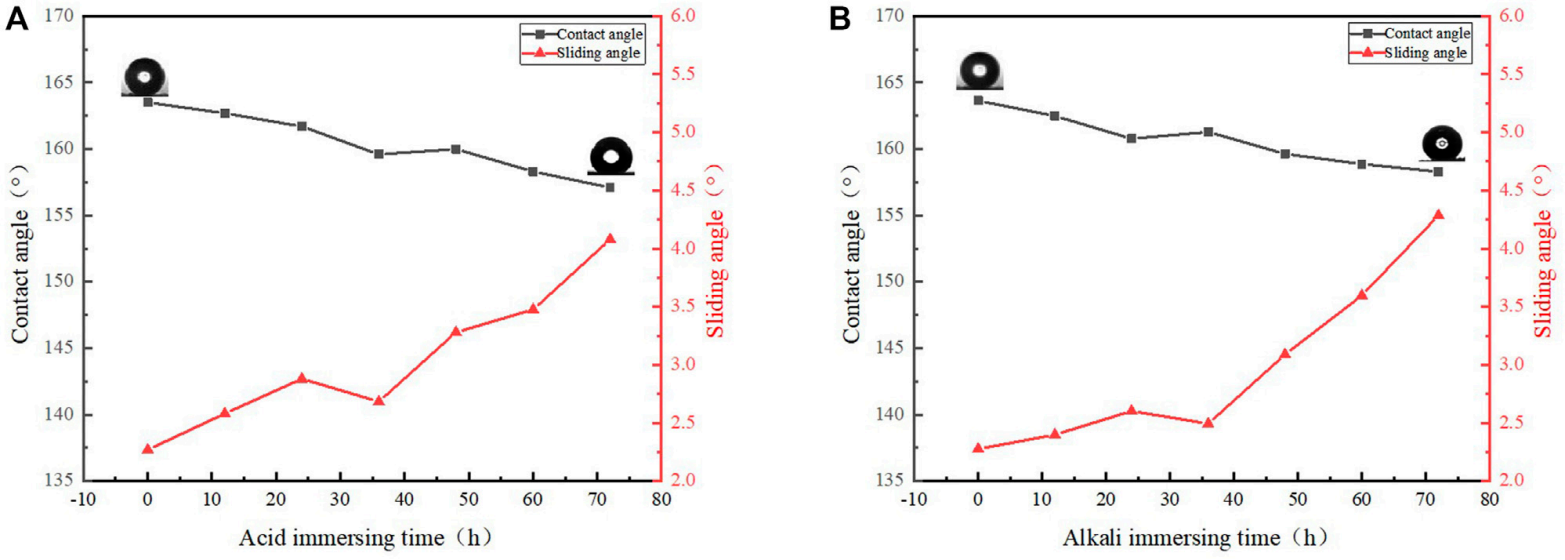
**(A)** Acid immersing result, **(B)** Alkali immersing result.

One of the important characteristics of oil-water separation materials is corrosion resistance. It is well known that copper is a metal with stable chemical properties. In the oil-water mixture often contains acidic or alkaline solution, which requires the product to have strong acid and alkaline resistance. The samples were immersed in sodium hydroxide solution and hydrochloric acid solution respectively. With the extension of time, WCA was measured every hour. It can be seen from Figure 8 that the WCA is reduced by a certain extent regardless of acid or alkali immersing, but the WCA is still above 150° and the WSA is still less than 5°. It can be concluded that the superhydrophobic copper mesh prepared by this method has good acid and alkali resistance.

### Oil and Water Separation

**FIGURE 9:**
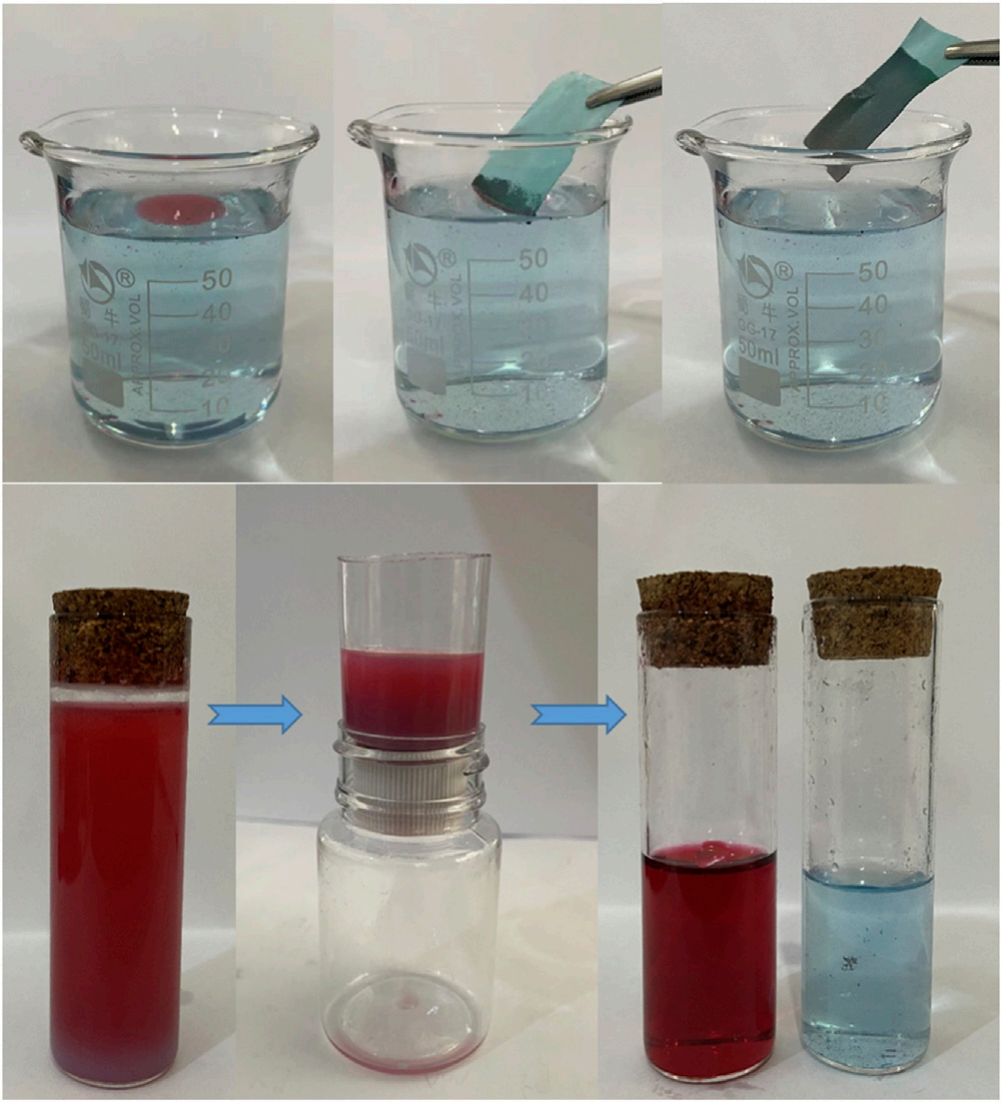
The process of oil floating adsorption and oil-water separation.

Figure 9 shows the process of oil floating adsorption and oil-water separation. The oil droplets on the water surface are absorbed by the copper mesh immersed in water (Supplementary Video S4. Floating adsorption video), and the adsorption efficiency is very high. In the process of oil-water separation, 20 ml water and 20 ml oil are dyed and mixed respectively, and the oil-water mixture is slowly poured into the device (Figure 9; Supplementary Video S5. Oil-water separation video). Superhydrophobic filter has excellent oil-water separation ability, which is its core content and plays a filtering role. After the oil-water mixture contacts with the filter screen, small oil droplets adsorb and penetrate on the surface of the filter screen, and the volume increases gradually. Finally, water is trapped on the surface of the filter screen after passing through the filter screen, until completely separated (Figure 9).

**FIGURE 10:**
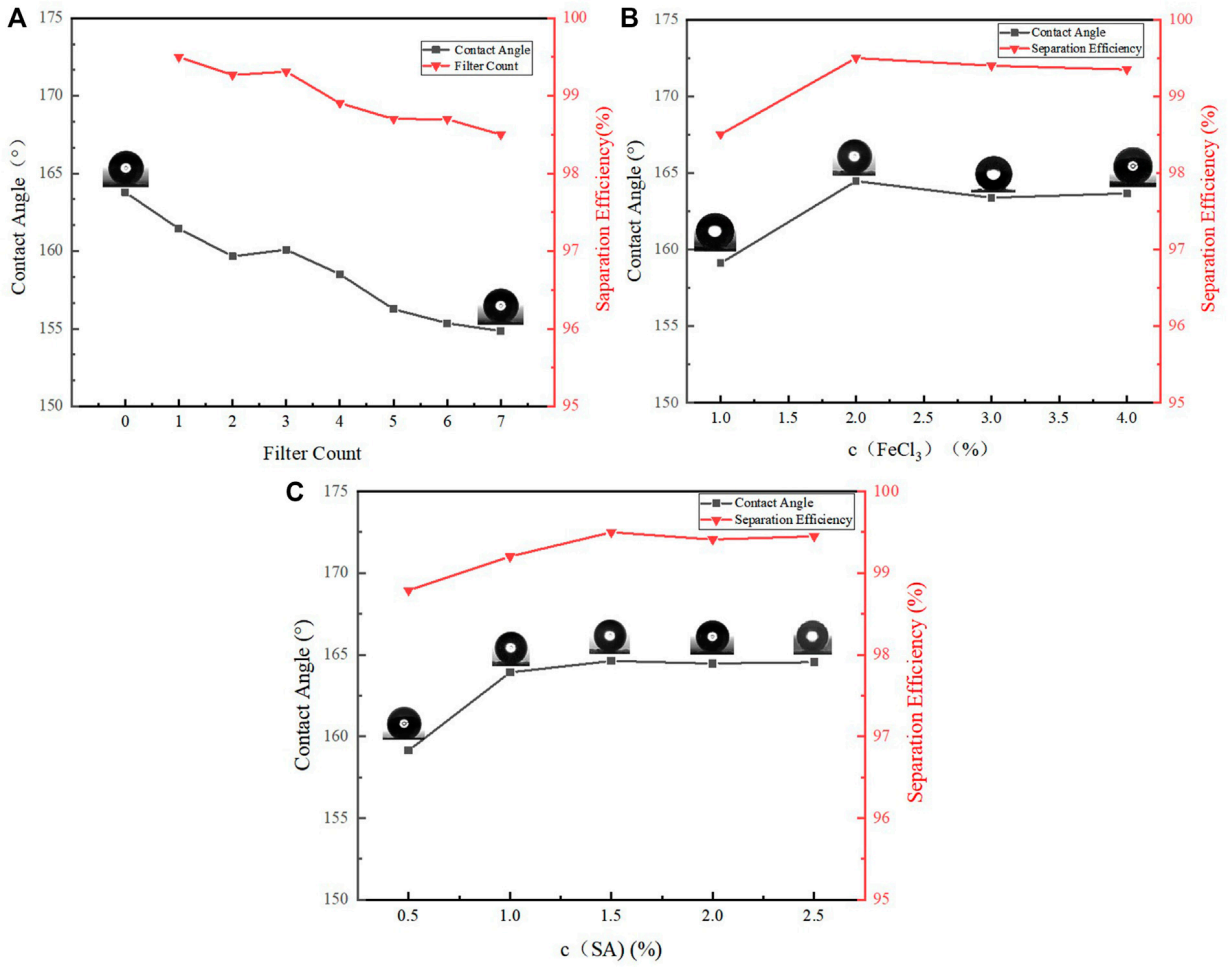
**(A)** Variation of oil-water separation efficiency and WCA with filter count, **(B)** Variation of oil-water separation efficiency and WCA with FeCl_3_ concentration, **(C)** Variation of oil-water separation efficiency and WCA with SA concentration.

In order to verify the durability of oil-water separation with copper mesh, seven oil-water separation experiments were carried out with the same copper mesh. The variation laws of WCA and oil-water separation efficiency are as Figure 10A.

Figure 10A shows the change of WCA and the efficiency of oil-water separation after repeated separate the oil-water mixture by the superhydrophobic copper mesh seven times. Although the WCA has a tendency to decrease after separations for 7 times, it is still greater than 150°, and the oil-water separation efficiency is also maintained above 98%.

Next, the effect of the concentration of FeCl_3_ solution on the WCA and oil-water separation efficiency was explored. Other conditions remained unchanged, the copper mesh of the same size was immersed into the FeCl_3_ solution with mass fraction of 1, 2, 3, 4 and 5%. Because the concentration of 5% is too high, the copper mesh is completely corroded, and only four pieces of copper mesh are finally obtained. The WCA of four kinds of copper mesh and the results of oil-water separation efficiency are shown in the Figure 10B.

It can be seen from the above Figure 10B that the WCA increases with the increase of ferric chloride concentration, and then decreases slightly. The WCA is the largest at 2%. As the copper mesh is completely corroded when the concentration is 5%, it is speculated that when the concentration is 3 and 4%, part of the pore diameter of the copper mesh is destroyed, making the roughness less than 2%. According to the oil-water separation experiment, except that the 1% aging rate is low, the oil-water separation efficiency of the later three groups of experiments is almost the same.

Finally, the influence of the concentration of SA in alcohol on the WCA and oil-water separation efficiency is explored. Other conditions remain unchanged. Soak the copper mesh with 0.5, 1, 1.5, 2 and 2.5% SA solution respectively to prepare five pieces of copper mesh. The WCA and oil-water separation efficiency of these copper meshes are shown in the Figure 10C.

Figure 10C shows that after the SA concentration increases to 1.5%, the SA concentration continues to increase, and the WCA basically does not change. the oil-water separation efficiency did not change significantly after 1.5%. When compared with 1.5%, the efficiency of 2 and 2.5% decreased slightly. It is speculated that the possible reason is that after the concentration increased to 1.5%, some products blocked the mesh, resulting in the decrease of oil-water separation efficiency.

## Conclusion

In summary, the copper mesh obtained by immersing method has obvious superhydrophobic property. The WCA of the copper mesh can reach about 160°, and the WSA is also kept below 5°. Due to the formation of micro nano block structure on the surface of copper mesh in the first step of the experiment, higher surface roughness is provided. Then it was immersed in ethanol solution of SA and copper stearate was grown on the surface *in-situ*.

The superhydrophobic copper mesh has excellent acid and alkali resistance. After the acid and alkali resistance test, the WCA of the copper mesh is still more than 150°, the WSA is also less than 5°. Meanwhile, it was found to be exceptionally efficient on oil-water separation, after seven cycles of separation, the oil-water separation efficiency remains above 97%. The reaction did not use fluoride-containing reagents or other large equipment, greatly saving research costs. It is believed that the superhydrophobic copper meshes fabricated by etching and SA surface in-suit growth method has promising practical applications on oil-water separation.

## Data Availability

The original contributions presented in the study are included in the article/Supplementary Material, further inquiries can be directed to the corresponding authors.
